# Painless Stanford type A aortic dissection presenting with isolated bilateral lower-limb neurological deficits: a case report

**DOI:** 10.1186/s43044-026-00799-4

**Published:** 2026-11-13

**Authors:** Dazheng Fang, Bin Zhao, Jinjie Li, Bing Wu

**Affiliations:** The Hospital of Shandong Provincial Corps of the Chinese People’s Armed Police Force, Jinan, China

**Keywords:** Aortic dissection, Stanford type A, Painless presentation, Lower-limb neurological deficits, Spinal cord malperfusion, Case report

## Abstract

**Introduction:**

Stanford type A aortic dissection (AAD) constitutes a life-threatening cardiovascular emergency. Painless AAD manifesting exclusively as isolated bilateral lower-limb neurological deficits represents an uncommon but recognised presentation of aortic dissection that is prone to misdiagnosis. This case report adds real-world clinical data regarding atypical AAD and delivers practical diagnostic and surgical insights for front-line clinicians.

**Main symptoms and important clinical findings:**

A 58-year-old Han Chinese female presented with acute-onset bilateral lower-limb numbness and weakness lasting one hour, in the absence of chest, back or abdominal pain. Physical examination uncovered an inter-arm systolic blood-pressure discrepancy together with bilateral lower-limb motor weakness (3/5). Emergency non-contrast chest-abdominal CT identified the aortic double-lumen sign and inward-displaced aortic wall calcified plaques, whereas bilateral distal peripheral pulses remained preserved.

**Main diagnoses, therapeutic interventions, and outcomes:**

Painless Stanford type A aortic dissection complicated by suspected transient spinal cord malperfusion was diagnosed. Following urgent inter-hospital transfer, the patient underwent emergency Bentall procedure combined with aortic arch reconstruction and frozen elephant-trunk graft implantation, with a distal landing zone in the proximal descending thoracic aorta. Her bilateral lower-limb neurological deficits had fully resolved by postoperative day 2. A residual chronic dissection with a thrombosed false lumen persisted distally below the stent-graft and remained stable without interval enlargement. Six-month follow-up confirmed complete functional recovery without residual neurological sequelae or proximal dissection recurrence.

**Conclusion:**

Undiagnosed painless Stanford type A aortic dissection presenting with isolated bilateral lower-limb neurological deficits is associated with substantial mortality. Routine bilateral upper-arm blood-pressure measurement and recognition of calcification displacement signs on non-contrast CT facilitate early clinical suspicion. Timely confirmation by CTA and urgent comprehensive surgical reconstruction are essential to reverse end-organ malperfusion and optimize neurological as well as survival outcomes.

**Supplementary Information:**

The online version contains supplementary material available at https://doi.org/10.1186/s43044-026-00799-4.

## Introduction

Acute aortic dissection (AD) is a catastrophic cardiovascular emergency associated with devastating early-phase mortality. Stanford type A aortic dissection (AAD), which involves the ascending aorta, mandates emergent surgical intervention to avert lethal complications including aortic rupture, cardiac tamponade, and end-organ malperfusion. Sharp, tearing chest or back pain constitutes the classic presenting symptom that triggers standard diagnostic work-up for aortic dissection in over 90% of affected patients [1].

Nevertheless, approximately 10%–15% of patients with AD exhibit atypical manifestations. Among this subgroup, purely painless presentations account for merely 5.0%–6.4% of all acute AD cases according to data from the authoritative International Registry of Acute Aortic Dissection (IRAD) [2]. Painless AAD independently predisposes to marked diagnostic delay and elevated in-hospital mortality, given that the absence of classic painful symptoms deviates substantially from the well-established clinical profile of AAD [3]. Such atypical phenotypes are frequently misdiagnosed as lumbar disc disease, primary spinal cord lesions, or acute cerebrovascular events within emergency-department settings [2, 4, 5].

Most documented cases of painless AAD are accompanied by syncope or cerebral ischemic events. By contrast, presentations in which isolated bilateral lower-limb neurological deficits constitute the sole clinical manifestation, without any painful or systemic symptoms, remain uncommon within the existing literature [5–7]. Notably, a large proportion of patients with undiagnosed painless AAD experience rapid clinical deterioration and sudden cardiac death, with definitive diagnoses established only at post-mortem autopsy; this observation underscores the exceedingly high risk inherent to this atypical disease variant [2]. This painless phenotype, characterized by isolated neurological impairment, poses considerable diagnostic challenges and represents an uncommon but recognised presentation of aortic dissection. Herein, we describe in detail the diagnostic workflow, standardized surgical management, and six-month long-term follow-up outcomes of this unusual case, with the aim of enhancing clinical awareness of atypical painless AAD, mitigating diagnostic delay, and improving prognosis among high-risk undiagnosed patients.

## Patient information

### De-identified patient characteristics

A 58-year-old Han Chinese female with no history of tobacco or alcohol consumption or other modifiable cardiovascular risk factors.

### Chief complaints and clinical presentation

The patient presented to the emergency department (ED) reporting one hour of acute spontaneous bilateral lower-limb numbness accompanied by progressive weakness. She denied all painful sensations (including chest, back, abdominal and lumbar pain), alongside dizziness, syncope, dyspnea, nausea and vomiting. No other systemic complaints were reported.

### Medical, family, and psychosocial history

She had no clinically documented history of hypertension, diabetes mellitus, coronary artery disease, or connective-tissue disorders. There was no family history of aortic dissection, aortic aneurysm, or hereditary vasculopathy. Although traditional cardiovascular risk factors were absent from her clinical record, preoperative CT imaging demonstrated mild aortic and coronary calcification, indicative of underlying subclinical atherosclerotic change.

### Prior interventions

No prior medical or surgical interventions relevant to vascular or neurological disease had been performed before admission.

## Clinical findings

### Vital signs

**Fig. 1:**
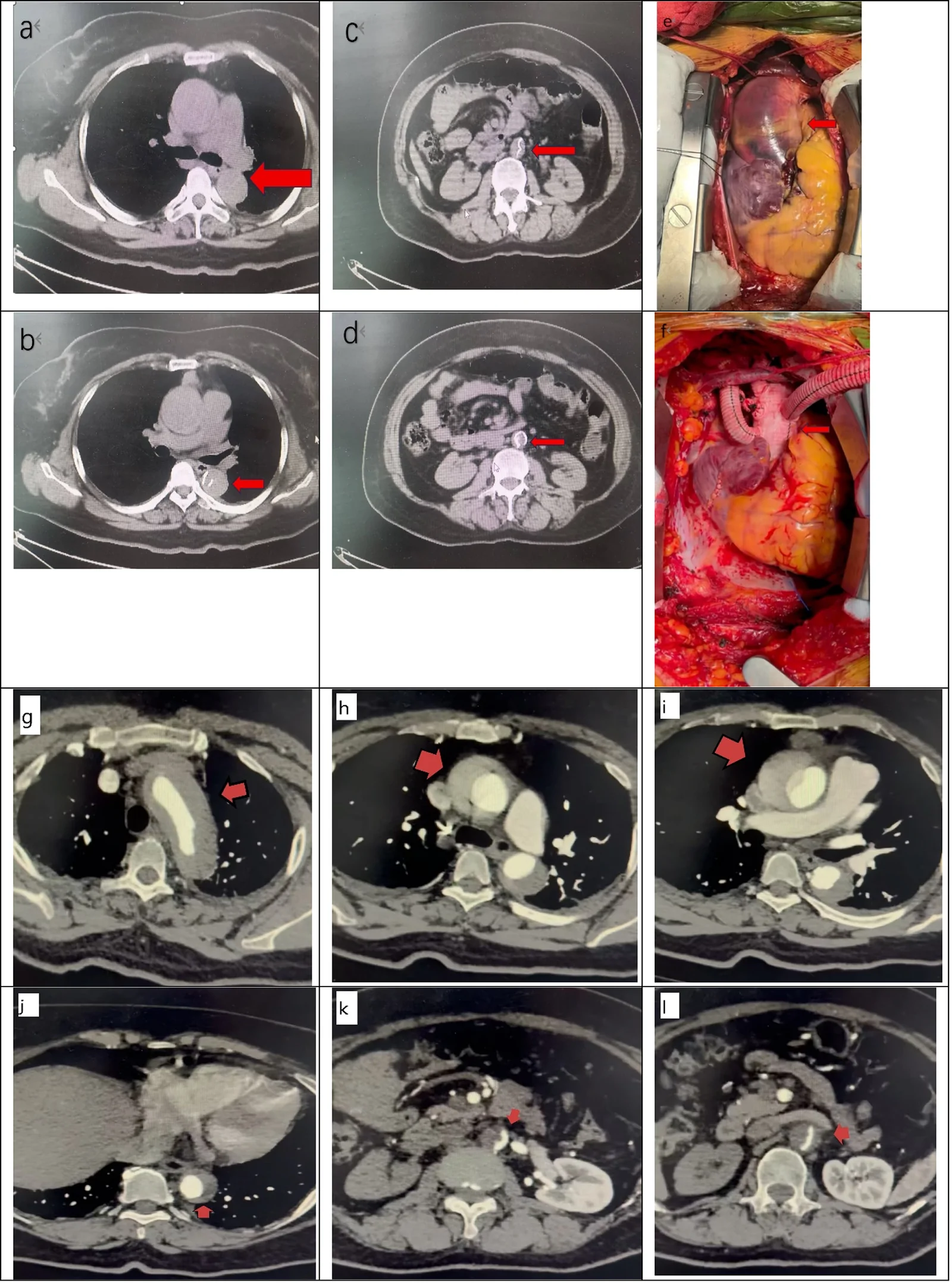
**a**: Non-contrast aortic CT scan demonstrating marked aortic dilatation and classic double-lumen sign, the characteristic imaging feature of aortic dissection (red arrow indicates the intimal flap). **b-d**: Non-contrast aortic CT showing inward displacement of aortic-wall calcified plaques (red arrows), a valuable indirect diagnostic clue for painless aortic dissection. **e**: Intraoperative photograph showing significant ascending aortic dilatation, a typical morphological feature of Stanford type A aortic dissection (red arrow). **f**: Intraoperative view after resection of native diseased aorta and implantation of composite Bentall graft combined with frozen elephant-trunk prosthesis (red arrow marks the graft anastomotic junction). **g-l**: Preoperative CTA showing that the dissection originated from an intimal tear at the aortic sinus, involving the entire ascending aorta, aortic arch, and proximal abdominal aorta. The aortic root and valve were directly involved, and the left renal artery was predominantly perfused via the false lumen (red arrow)

Baseline vital signs upon ED presentation were as follows: body temperature 36.5 ℃, pulse rate 88 beats per minute, respiratory rate 20 breaths per minute, and room-air oxygen saturation of 99%. A prominent inter-arm blood-pressure discrepancy was detected: 123/45 mmHg in the left arm versus 97/41 mmHg in the right arm, accompanied by widened pulse pressure. Tachycardia and hypotension were absent (Fig. 1).

### Physical examination

- **Neurological assessment**: The patient remained fully alert, oriented and verbally responsive, with intact cranial-nerve function. Upper-limb motor and sensory function was bilaterally normal (5/5 strength). Marked reduction in bilateral lower-limb motor strength (3/5) was noted together with reduced muscle tone. Patellar tendon reflexes were bilaterally attenuated, and no pathological reflexes could be elicited. Distal-predominant numbness affected both lower extremities; gait disturbance and sphincter dysfunction (urinary or fecal) were absent.
- **Cardiopulmonary assessment**: Cardiac auscultation revealed regular rhythm. Despite widened pulse pressure, no cardiac murmurs, gallops, or pericardial friction rubs were audible. Pulmonary auscultation demonstrated clear bilateral lung fields without rales or wheezes.
- **Abdominal assessment**: The abdomen was soft and non-tender, without rebound tenderness or guarding. No abdominal mass or organomegaly was palpable, and shifting dullness was negative.
- **Extremity assessment**: There was no lower-limb edema, cyanosis, or cold extremities. Importantly, bilateral dorsalis pedis and posterior tibial arterial pulses were fully palpable and symmetrical. Although palpable distal pulses argued against large-vessel peripheral arterial occlusion, palpable pulses alone cannot definitively exclude limb malperfusion; the deficit was therefore considered most likely to reflect presumed spinal cord hypoperfusion rather than peripheral limb ischaemia.

Examination of the skin, lymphatic system, and genitourinary tract yielded unremarkable findings.

## Timeline

1. 16:54, 19 November 2025: At rest, the patient developed acute, entirely painless bilateral lower-limb numbness and weakness, with no spontaneous symptom relief and no self-administered treatment.
2. 17:54, 19 November 2025: The patient presented to a local ED. Bedside physical examination identified inter-arm blood-pressure asymmetry and bilateral lower-limb motor weakness, prompting urgent vascular imaging.
3. 18:10, 19 November 2025: Emergency non-contrast head and chest-abdominal CT scans were acquired. Typical indirect signs of aortic dissection, including an intimal flap (double-lumen sign) and inward displacement of aortic wall calcified plaques, were clearly visualized throughout the thoracic and abdominal aorta. Non-contrast head CT demonstrated no acute intracranial haemorrhage or established cerebral infarction, although an early non-contrast study cannot definitively exclude acute ischaemic stroke.
4. 18:30, 19 November 2025: A provisional diagnosis of painless Stanford type A aortic dissection was established. Standard emergency bridging therapy was initiated to stabilize hemodynamics and reduce aortic wall shear stress.
5. 19:00, 19 November 2025: The patient was emergently transferred to a tertiary cardiovascular-surgery center for definitive diagnosis and operative management.
6. Post-transfer admission: Whole-aorta contrast-enhanced computed-tomography angiography (CTA) was performed to delineate the full anatomical extent of dissection. Preoperative transoesophageal echocardiography (TEE) could not be performed because a coexisting oesophageal stricture precluded passage of the probe. Only a rapid bedside transthoracic echocardiogram (TTE) was obtained in the emergency setting, and no formal archived echocardiographic report or images were available for subsequent review. The anatomical boundaries of the dissection were therefore characterised by CTA supplemented by systematic intraoperative macroscopic inspection.
7. 22:00, 19 November 2025: Emergency open complex aortic surgery was performed under general anesthesia and cardiopulmonary bypass (CPB), comprising Bentall procedure, aortic-arch reconstruction, and proximal descending-aorta frozen elephant-trunk graft implantation; no intraoperative complications occurred.
8. Postoperative day (POD) 2: Bilateral lower-limb numbness and weakness had completely resolved, with restoration of full bilateral motor strength (5/5).
9. Post-discharge follow-up: The patient experienced an uneventful inpatient recovery and was successfully discharged. Owing to residential relocation and suboptimal adherence to scheduled in-person follow-up appointments, part of the six-month follow-up dataset was obtained via standardized telephone interview. Serial imaging and neurological assessments confirmed preserved graft patency, absence of dissection recurrence, and complete resolution of neurological deficits.

## Diagnostic assessment

### Diagnostic testing

1. **Initial non-contrast CT imaging**: Emergency non-contrast chest-abdominal CT demonstrated aortic dilatation (maximum diameter: 5.6 cm), a diffuse aortic intimal dissection flap, and characteristic inward displacement of aortic-wall calcified plaques extending from the thoracic aorta to the left renal hilum. These pathognomonic indirect imaging markers delivered critical early diagnostic clues permitting rapid preliminary diagnosis within resource-constrained primary-care emergency settings. Incidental findings encompassed mild coronary calcification and a small benign pericardial effusion without tamponade physiology.
  Following transfer to the tertiary center, whole-aorta CTA clarified the detailed anatomical features and total extent of dissection. The primary intimal entry tear originated precisely at the aortic sinus; dissection propagated continuously to involve the aortic root, entire ascending aorta, complete aortic arch, descending thoracic aorta, and proximal abdominal aorta. The distal extent of the dissection reached the level of the left renal artery. The dissection flap directly involved and distorted the aortic root and aortic-valve leaflets, and marked leaflet malcoaptation was identified on intraoperative surgical inspection. Because the coexisting oesophageal stricture precluded TEE and no formal echocardiographic report or images were archived, no quantitative echocardiographic assessment of aortic regurgitation — such as vena contracta width, regurgitant jet area, or effective regurgitant orifice area — could be performed or retrieved; the anatomical severity of leaflet involvement was therefore established by direct surgical inspection rather than by echocardiographic measurement. The right-coronary-artery ostium lay in immediate proximity to the primary entry tear and was partially covered by the false lumen. The origins of the brachiocephalic trunk and left common carotid artery, located within the aortic arch, were both encroached upon by the intimal flap. Within the abdominal aorta, the left-renal-artery ostium was predominantly perfused by the false lumen and therefore incorporated within the dissection territory; this distal segment lay beyond the intended distal landing zone of the planned proximal aortic repair. No definitive radiological evidence of spinal-arterial occlusion, stenosis, or spinal-cord infarction was detected on comprehensive preoperative CTA.
  Notably, formal spinal magnetic-resonance imaging (MRI) or spinal vascular angiography was not performed, for three well-grounded clinical reasons. First, although preoperative whole-aorta CTA revealed neither direct nor indirect imaging stigmata of spinal vascular malperfusion nor structural spinal-cord injury, CTA cannot reliably exclude segmental spinal-artery compromise. Second, the patient’s acute bilateral lower-limb neurological symptoms resolved rapidly and completely within 48 h of surgical aortic revascularization, a course consistent with, but not proof of, reversible functional impairment rather than irreversible organic spinal-cord damage. Third, after thorough clinical counseling, the patient declined further spinal imaging on personal grounds. Accordingly, bilateral lower-limb neurological deficits were comprehensively attributed clinically to suspected transient spinal-cord malperfusion secondary to aortic dissection, rather than radiologically proven spinal-cord infarction; this conclusion synthesizes clinical presentation, imaging findings, rapid postoperative recovery, and intraoperative observations.
2. **Physical-examination clues**: The constellation of unexplained inter-arm blood-pressure discrepancy, isolated bilateral lower-limb sensorimotor dysfunction, attenuated patellar reflexes, and intact symmetrical distal peripheral pulses furnished vital bedside diagnostic hints. Palpable, symmetrical distal limb pulses argued against large-vessel peripheral arterial occlusion, although they do not definitively exclude limb malperfusion; the findings were considered most consistent with presumed transient spinal hypoperfusion rather than peripheral limb ischaemia.
3. **Laboratory testing**: Emergency routine hematology, coagulation studies, and D-dimer assays were obtained. No laboratory abnormalities could account for acute neurological or ischemic events attributable to alternative etiologies.

### Diagnostic challenges

1. **Atypical clinical presentation**: Purely isolated neurological symptoms without classic aortic-dissection-related pain readily mimic primary spinal or neurological disease, predisposing to initial misdiagnosis in emergency practice.
2. **Absence of conventional high-risk factors**: No documented hypertension, diabetes mellitus, or familial vasculopathy was present, which substantially lowered preoperative clinical suspicion for AAD, notwithstanding subclinical atherosclerotic calcification visualized on imaging.
3. **Limitations of initial non-contrast CT**: Within resource-limited community emergency departments, non-contrast CT frequently represents the first accessible imaging modality. Although it yields valuable suggestive indirect markers for aortic-dissection screening (calcified-plaque displacement, double-lumen sign), it cannot fully characterize true- versus false-lumen hemodynamics, branch-vessel perfusion status, or precise dissection boundaries, nor can it definitively confirm or rule out AD. Contrast-enhanced CTA remains the gold-standard investigation for definitive anatomical diagnosis of AD.

### Final diagnosis and differential diagnoses

#### Final diagnosis

Painless Stanford type A aortic dissection with suspected transient spinal-cord malperfusion presenting with isolated bilateral lower-limb neurological deficits.

#### Differential diagnoses

1. **Lumbar intervertebral-disc herniation**: This condition commonly produces lower-limb numbness and weakness but is typically accompanied by lumbago and radicular pain; furthermore, it cannot explain the observed inter-arm blood-pressure discrepancy.
2. **Acute ischemic stroke**: May produce unilateral or bilateral limb neurological deficits; however, a normal early non-contrast head CT together with the absence of cerebrovascular risk factors rendered this diagnosis unlikely, although an early non-contrast CT cannot definitively exclude acute ischaemic stroke.
3. **Primary spinal-cord lesions**: Including myelitis and spontaneous spinal-cord infarction, which can cause bilateral lower-limb sensorimotor impairment. Nevertheless, these entities fail to account for extensive aortic-dissection changes on imaging, and confirmatory spinal imaging was not acquired.
4. **Acute peripheral-arterial occlusion**: May produce limb ischemic dysfunction; however, although bilateral lower-limb peripheral pulses remained symmetrical and palpable throughout admission, palpable pulses alone cannot definitively exclude limb malperfusion, and this diagnosis was therefore regarded as unlikely rather than formally excluded. The deficit was attributed to presumed spinal-cord hypoperfusion rather than to peripheral limb ischaemia.

### Prognosis

Untreated acute Stanford type A aortic dissection carries a dismal prognosis, with an estimated mortality of approximately 1% per hour during the acute phase after symptom onset. Most patients with undiagnosed painless AAD succumb rapidly, with diagnoses only established post-mortem [2]. The present patient harbored hyperacute extensive AAD involving the aortic root, arch, and thoracoabdominal aorta complicated by suspected spinal-cord malperfusion, representing a high-risk surgical case. Fortunately, timely diagnosis and urgent complete aortic reconstruction yielded favorable short- and intermediate-term outcomes without permanent neurological disability or fatal complications.

## Therapeutic intervention

### Therapeutic modalities

1. **Preoperative emergency bridging therapy**: Continuous invasive hemodynamic monitoring, low-flow nasal oxygen supplementation (2 L/min), intravenous sodium nitroprusside for blood-pressure control, and metoprolol for heart-rate reduction. Target hemodynamic parameters were systolic blood-pressure 100–120 mmHg and heart rate 60–80 beats per minute, with the objective of reducing aortic-wall shear stress and preventing preoperative aortic rupture or worsening malperfusion.
2. **Definitive emergency surgical treatment**: Complex combined aortic-repair surgery incorporating Bentall procedure (mechanical composite aortic-valve and aortic-root replacement), total aortic-arch reconstruction, and proximal descending-aorta frozen elephant-trunk stent-graft implantation performed under CPB. This surgical strategy constitutes established, mature standard-of-care therapy for complex Stanford type A dissection affecting the aortic root and arch. Intraoperative systematic inspection confirmed satisfactory visceral and left-renal-artery perfusion without irreversible end-organ malperfusion or ischemic injury [8].
3. **Postoperative supportive treatment**: Perioperative antibiotic prophylaxis, fluid and nutritional support, standardized neurological rehabilitation, and targeted anticoagulation therapy.

### Treatment administration and detailed operative findings

1. **Bridging-therapy implementation**: All vasoactive agents were delivered via continuous intravenous infusion with real-time hemodynamic titration. Vital signs were monitored every 5 min during emergency stabilization to sustain hemodynamic stability prior to definitive surgery.
2. **Detailed operative parameters and findings**: Median sternotomy was performed. Cardiopulmonary bypass was established through ascending-aortic and two-stage right-atrial cannulation, with the right axillary artery additionally cannulated through an 8-mm prosthetic side graft to deliver unilateral antegrade cerebral perfusion during circulatory arrest. Correct placement within the true lumen was confirmed by the return of pulsatile flow, satisfactory systemic perfusion pressure, and direct intraoperative inspection of the cannulated aortic segment. Intraoperative transoesophageal echocardiography was not performed, as the coexisting oesophageal stricture precluded probe insertion; intraoperative assessment of the aortic valve and root was therefore made by direct surgical inspection. Key intraoperative metrics included a total operative duration of 4 h, a total cardiopulmonary bypass duration of 90 min, an aortic cross-clamp time of 72 min, and a moderate-hypothermic circulatory-arrest time of 22 min at a nasopharyngeal temperature of 25 °C. A 24-mm mechanical composite Bentall graft was implanted with precise re-implantation of both coronary-artery ostia; the total aortic arch was replaced with a 28-mm four-branch Dacron arch graft, and the arch vessels were reconstructed using the en-bloc (island) technique. A 26 × 100-mm frozen elephant-trunk stent-graft was deployed into the proximal descending thoracic aorta. Intraoperative inspection fully corroborated preoperative CTA findings: the primary intimal tear arose at the aortic sinus, inducing marked aortic-root dilatation and severe aortic-valve leaflet malcoaptation. Dissection extensively involved the entire ascending aorta, full aortic arch, and proximal descending aorta. The frozen elephant-trunk stent-graft was deployed within the proximal descending thoracic aorta to treat the distal dissection pathology, abolish false-lumen perfusion within the stent-grafted segment, and stabilise the aortic wall at this level. Isolated ascending-aorta replacement was precluded by extensive arch and descending-aorta involvement; the comprehensive combined aortic-repair strategy was adopted to resect the diseased aortic segments within the proximal repair zone and eliminate false-lumen perfusion at the level of the stent-graft, while deliberately leaving a residual chronic dissection in the distal descending and proximal abdominal aorta below the stent-graft. A staged approach was adopted in preference to extended thoracoabdominal repair, in order to limit operative risk, with the residual distal dissection to be managed by surveillance and a low threshold for distal aortic re-intervention should progression occur. Intraoperative visual and Doppler assessment confirmed patency and adequate perfusion of the left renal artery without definite ischemic lesions.
3. **Postoperative medication and rehabilitation**: Intravenous antibiotics were administered for 7 days for standard perioperative infection prophylaxis. Oral warfarin anticoagulation (target INR 2.0–3.0) was prescribed lifelong postoperatively; this was strictly indicated by mechanical aortic-valve prosthesis implantation, which mandates indefinite anticoagulation, rather than constituting routine adjuvant postoperative therapy. Early bedside neurological rehabilitation and lower-limb functional training were commenced on POD 2 immediately following neurological recovery to facilitate prompt functional restoration.

Discharge medications: A complete, structured medication regimen was prescribed at discharge: warfarin sodium 2.5 mg once daily (dose titrated to maintain an INR of 2.0–3.0; lifelong therapy), metoprolol tartrate 25 mg twice daily (target resting heart rate 60–80 beats per minute), valsartan 80 mg once daily (target blood pressure < 120/80 mmHg), atorvastatin calcium 20 mg once nightly, enteric-coated aspirin 100 mg once daily after meals, and pantoprazole 40 mg once daily before breakfast (continued for 1–3 months as clinically indicated). The complete bilingual discharge medication list, together with monitoring and follow-up instructions, is provided as a supplementary file.

### Modifications to therapeutic strategy and rationale

1. **Transition from medical stabilization to urgent surgery**: Medical therapy serves only as temporary bridging for AAD. Emergent resection of diseased aortic segments together with vascular reconstruction represents the sole definitive life-saving intervention for preventing aortic rupture and cardiac tamponade.
2. **Perioperative medication conversion**: Following postoperative hemodynamic and gastrointestinal stabilization, intravenous agents were switched to oral formulations to mitigate catheter-associated infection risk and facilitate early rehabilitation and hospital discharge.
3. **Early neurological rehabilitation**: Preoperative neurological dysfunction was presumed secondary to transient spinal-cord hypoperfusion. Early mobilization and standardized rehabilitation promote neurological functional recovery and avert persistent limb impairment.

## Follow-up and outcomes

### Clinician-reported and patient-reported outcomes

#### Postoperative inpatient outcomes

The postoperative course was uncomplicated. ICU stay spanned 3 days, and total hospital stay was 10 days. Mechanical ventilation was discontinued on POD 1 once spontaneous breathing had fully returned. No major postoperative complications occurred, including surgical-site infection, mediastinitis, graft leakage, bleeding requiring re-exploration, renal impairment, stroke, or cardiac adverse events. Renal function remained within reference ranges throughout hospitalization and follow-up.

1. **Clinician-assessed outcomes**: Complete resolution of bilateral lower-limb numbness and weakness was achieved on POD 2, accompanied by full restoration of lower-limb sensorimotor function. The inter-arm blood-pressure discrepancy resolved completely post-operatively. One-month and three-month in-person follow-up assessments were completed as scheduled. Due to residential relocation and poor adherence to subsequent in-person visits, six-month follow-up information was partially collected by standardized telephone interview. All available follow-up data confirmed stable vital signs, normal neurological examination, patent prosthetic grafts, and absence of dissection recurrence or stenosis. At the most recent follow-up, the patient retained full capacity for activities of daily living.

2. **Patient-reported outcomes**: The patient reported complete resolution of all preoperative neurological symptoms, with independent ambulation and self-care capability. She described good adherence to postoperative warfarin anticoagulation and rehabilitation guidance throughout follow-up, with satisfactory overall recovery.

### Follow-up investigations

1. **Imaging follow-up**: One-month postoperative CTA demonstrated unobstructed Bentall composite graft and frozen elephant-trunk prosthesis, no new dissection lesions, and normal perfusion of the aorta and its branch vessels. A residual chronic dissection with a thrombosed false lumen persisted in the distal descending and proximal abdominal aorta below the distal end of the stent-graft, including the level of the left renal artery, with restoration of antegrade true-lumen perfusion to the left renal artery. Subsequent 3-month and 6-month thoracic-abdominal CT confirmed stable aortic morphology. An incidental small left-adrenal space-occupying lesion showed no interval enlargement and required no active clinical intervention.

2. **Laboratory follow-up**: Serial complete blood counts, liver-renal biochemistry, and coagulation parameters remained within normal limits. Postoperative D-dimer concentrations normalized, and anticoagulation maintained INR consistently within the target therapeutic window.

3. **Neurological follow-up**: Serial neurological examinations documented normal bilateral lower-limb muscle strength, tendon reflexes, and sensation without pathological reflexes, consistent with durable complete neurological recovery and absence of symptom relapse.

### Treatment adherence and tolerability

1. **Adherence**: The patient fully complied with all emergency therapeutic and surgical measures during hospitalization. Following discharge, adherence to oral warfarin anticoagulation and rehabilitation training remained satisfactory. Partial loss of complete in-person follow-up data is attributable exclusively to residential relocation and suboptimal long-term appointment attendance and does not reflect inadequate short-term treatment compliance.

2. **Tolerability**: Preoperative bridging medical therapy was well tolerated without hypotension or bradycardia. Complex aortic surgery was accomplished successfully without intraoperative adverse events. Post-discharge medications and rehabilitation training were well tolerated, with no drug-related adverse effects or rehabilitation-associated discomfort.

### Adverse and unexpected events

No adverse events arose during preoperative hemodynamic stabilization or inter-hospital transfer. No catastrophic intraoperative hemorrhage, aortic rupture, or CPB-related complications were noted. Throughout hospitalization and the six-month follow-up interval, no major postoperative adverse events were recorded, including surgical infection, prosthetic-graft malfunction, cerebrovascular accident, recurrent organ malperfusion, multi-organ failure, or sudden death.

## Discussion

The present report describes an unusual and clinically instructive case of painless Stanford type A aortic dissection in which isolated bilateral lower-limb neurological deficits constituted the sole presenting manifestation, without classic chest or back pain. This highly atypical phenotype generates substantial diagnostic difficulty and is readily misdiagnosed as primary neurological or spinal pathology. The following discussion systematically analyzes the case’s distinctive clinical features, diagnostic pitfalls, plausible pathophysiological hypotheses, evidence-based surgical rationale, study strengths and limitations, and clinical implications, grounded strictly in verified clinical observations and authoritative published evidence. Rather than emphasising the rarity of this presentation, we frame it as an uncommon but recognised manifestation of aortic dissection, the clinical value of which lies in its diagnostic and therapeutic implications.

### Clinical features and diagnostic value of this uncommon presentation

Classic Stanford type A aortic dissection is dominated by acute severe tearing chest or back pain, which serves as the cardinal diagnostic clue enabling early clinical recognition [2]. According to IRAD registry data, painless AAD accounts for merely 5.0%–6.4% of all acute AD episodes and independently correlates with diagnostic delay and elevated early mortality [2, 3]. Most previously published painless-AAD cases present with syncope or acute cerebral ischemic events; presentations characterized by completely painless onset, absence of systemic symptoms, and isolated bilateral lower-limb neurological deficits as the sole clinical feature are infrequently reported in the contemporary literature [6]. Moreover, most patients with undiagnosed atypical painless AAD experience rapid clinical deterioration and sudden death, with diagnoses confirmed only post-mortem, yielding very low survival among such misdiagnosed cases [2]. By contrast, timely surgical intervention in our patient permitted rapid full neurological recovery and favorable intermediate-term prognosis, rendering this case a useful clinical reference.

Three key clinical screening steps underpinned successful early diagnosis in this unusual scenario and represent its major clinical highlights. First, routine bilateral upper-extremity blood-pressure measurement upon admission identified subtle inter-arm blood-pressure discrepancy, an early warning sign pointing toward central aortic vasculopathy and preventing misdiagnosis as primary spinal disease. Second, characteristic inward displacement of aortic-wall calcified plaques seen on initial non-contrast CT delivered rapid, reliable indirect imaging evidence permitting preliminary diagnosis within resource-limited community emergency settings, securing precious time for definitive subsequent therapy. Third, although palpable bilateral distal peripheral pulses do not definitively exclude limb malperfusion, their preservation made large-vessel peripheral arterial occlusion unlikely and helped clarify the differential-diagnostic pathway at an early disease stage.

### Presumed pathophysiological mechanism underlying isolated lower-limb neurological deficits and justification for omitted spinal imaging

Bilateral lower-limb sensorimotor impairment in this case is clinically hypothesized to arise from transient reversible spinal-cord malperfusion secondary to aortic dissection, rather than irreversible peripheral-limb ischemia or organic spinal-cord infarction. The principal supporting clinical evidence consists of persistently intact, symmetrical distal peripheral pulses bilaterally; these argue against, but do not definitively exclude, large-vessel peripheral arterial occlusion, since palpable pulses alone cannot rule out limb malperfusion.

We postulate that the extensive aortic-dissection flap dynamically compressed or intermittently obstructed segmental spinal-artery ostia originating from the descending aorta, producing reversible spinal-cord hypoperfusion and acute myelopathic symptoms [3, 9]. This pathophysiological construct aligns with previously described aortic-dissection-associated spinal malperfusion syndrome [9]. It merits emphasis that formal spinal MRI or spinal vascular angiography was not performed for three clinically rigorous reasons: first, comprehensive preoperative whole-aorta CTA demonstrated no radiological markers of spinal-vascular stenosis, occlusion, or structural spinal-cord injury; second, complete resolution of neurological symptoms within 48 h of aortic revascularization was consistent with, but did not prove, a functional and reversible impairment; third, after full clinical explanation, the patient explicitly declined additional spinal imaging for personal reasons. Spinal-cord malperfusion in this case therefore represents a rigorous clinical inference synthesized from multiple datasets, rather than speculative conjecture [3].

In addition, several plausible mechanistic hypotheses may explain the fully painless phenotype: comparatively slow dissection propagation velocity reducing mechanical stretch and nociceptive stimulation of aortic-adventitial nerve endings, inter-individual variability in systemic pain-perception thresholds, and masking of subtle aortic nociceptive signals by concurrent acute spinal neurological dysfunction. These interpretations are clinically reasonable and consistent with current literature regarding mechanisms underlying painless AAD.

### Literature comparison and clinical value of the case

Existing literature documents limited numbers of atypical painless-AAD presentations, predominantly syncope, acute stroke, and unilateral limb weakness [2, 4, 5, 7]. Campa et al. reported individual AAD cases manifesting as acute paraplegia, yet most of those patients had associated vague systemic symptoms or mild atypical pain [6, 10]. The distinguishing features of the present case are its completely painless acute onset, pure bilateral lower-limb neurological deficits without additional systemic symptomatology, rapid full neurological recovery following emergency surgery, and favorable long-term prognosis. It further expands the known phenotypic spectrum of atypical painless AAD. A condensed item-by-item comparison of the present case with these published case reports is presented in Table 1; a detailed comparison with the case reports, review articles, and cohort studies discussed above, including verification of all cited references, is provided in Additional file 1.

**Table 1:** Comparison of the present case with key published case reports of painless aortic dissection

Variable	Present case	Ismail 2025 [4]	Hattab 2022 [5]	Campa 2023 [6]	Sakurai 2025 [7]
Age / sex	58 y / female	59 y / male	66 y / male	42 y / male	84 y / female
Pain at onset	Absent throughout	Absent	Absent (asymptomatic)	Chest pain radiating to back	Absent
Principal manifestation	Isolated bilateral lower-limb numbness and weakness	Syncope, right upper-limb weakness, slurred speech	No symptoms (chronic dissection)	Sudden paraplegia with sensory loss	Transient bilateral lower-limb weakness with bradycardia
Presumed mechanism of ischaemia	Transient spinal-cord hypoperfusion	Multi-organ ischaemia	None	Suspected spinal-cord ischaemia	Spinal-cord hypoperfusion due to hypotension
Surgical strategy	Bentall + total arch replacement + frozen elephant trunk	Emergency aortic repair	Referred for surgery	Arch and Bentall repair	Valve-sparing ascending aortic replacement
Neurological recovery	Complete by POD 2	Good	Not applicable	No recovery	Complete

A more detailed item-by-item comparison, including the review articles, cohort studies and verification of all cited references, is provided in Additional file 1. POD, postoperative day

In contrast to many historical AAD cases complicated by spinal malperfusion, which are characterized by irreversible neurological deficits and poor prognosis, our patient achieved complete neurological functional restoration within 48 h of surgical aortic revascularization. This observation supports the view that timely, standardized surgical intervention can reverse presumed transient spinal-cord hypoperfusion and avert permanent neurological disability [9]. This case furnishes a noteworthy positive prognostic reference for high-risk painless AAD complicated by suspected spinal malperfusion and carries substantial clinical educational value.

### Standardized surgical strategy and anticoagulation rationale

The surgical approach implemented conforms fully to contemporary clinical guidelines for complex Stanford type A aortic dissection. Given extensive dissection involving the aortic sinus, aortic root, aortic valve, entire aortic arch, and proximal descending aorta, combined Bentall procedure + aortic-arch reconstruction + frozen elephant-trunk graft implantation represents the most appropriate and clinically mature comprehensive surgical strategy. Because the aortic root was directly involved by the dissection and was dilated, and because the dissected root tissue was markedly fragile in the acute setting, composite root replacement (Bentall procedure) was preferred over aortic-valve resuspension or a root-preserving repair. Even in the absence of archived echocardiographic evidence of severe aortic regurgitation, a valve-sparing or root-preserving approach would have carried an appreciable risk of late root rupture and re-dissection in this acutely dissected, structurally compromised aortic root. This operation achieves resection of the diseased aortic segments within the proximal repair zone, repair of arch-vessel pathology, obliteration of false-lumen perfusion within the stent-graft segment, and restoration of physiological perfusion to the aortic arch and its branch vessels. It should be emphasised that the distal abdominal aortic segment harbouring the residual dissection lies beyond the distal extent of this repair and requires continued imaging surveillance, as the presence of a residual distal false lumen is an inherent feature of proximal aortic repair for extensive type A dissection. Intraoperative assessment of left-renal-artery perfusion excluded irreversible ischemic injury, concordant with preserved postoperative renal function [8].

Postoperative lifelong warfarin anticoagulation targeting an INR of 2.0–3.0 was mandated exclusively by mechanical aortic-valve prosthesis implantation and does not represent routine therapy for aortic-dissection patients. This practice adheres strictly to standardized cardiac-surgery pharmacotherapy specifications.

### Follow-up limitations and objective explanation

Due to postoperative residential relocation and suboptimal long-term adherence to scheduled in-person follow-up appointments, a subset of six-month intermediate-term follow-up data were collected by standardized telephone interview instead of in-person physical examination and imaging review. This objective circumstance generates partial follow-up-dataset limitations, which are stated transparently in the study-limitations section without data exaggeration or omission, safeguarding study authenticity and methodological rigor.

### Study strengths and limitations

#### Strengths

This case report describes an uncommon but clinically important phenotype of completely painless Stanford type A aortic dissection with isolated neurological manifestations, augmenting real-world clinical data concerning atypical AAD. Standardized bedside blood-pressure screening, valuable indirect non-contrast-CT imaging clues, timely standardized comprehensive surgical intervention, and favorable long-term prognosis deliver practical diagnostic, therapeutic, and follow-up references for front-line clinicians.

#### Limitations

First, this constitutes a single-patient retrospective case report; accordingly, its conclusions cannot be generalized to all patients with AAD. Second, definitive spinal imaging was not performed; spinal-cord malperfusion represents an integrated clinical inference rather than a radiologically confirmed diagnosis. Third, initial emergency diagnosis depended upon non-contrast CT with inherent diagnostic constraints. Fourth, portions of six-month follow-up data were obtained via telephone interview, conferring lower objectivity relative to complete in-person follow-up. Fifth, echocardiographic documentation was incomplete: TEE was precluded by a coexisting oesophageal stricture, only a bedside TTE was performed and no formal images were archived, and the anatomical severity of aortic-valve involvement was therefore based on intraoperative inspection rather than formal echocardiographic measurement. Sixth, because the emergency operation was performed at another institution and policy-related constraints limited access to the original records, part of the operative and follow-up information was retrieved through telephone contact with the treating surgeons, so some operative detail remains incomplete. These limitations are acknowledged transparently, and the findings should be interpreted in the light of the single-case, retrospective design and the incomplete availability of source data.

## Supplementary Information

Below is the link to the electronic supplementary material.

Supplementary Material 1

Supplementary Material 2

## Data Availability

No datasets were generated or analysed during the current study.

## Declarations

### Patient perspective

I am a 58-year-old female who previously considered myself in good health without chronic medical conditions. On 19 November 2025, while resting, I suddenly developed numbness and weakness affecting both lower legs, with no chest, back, or lumbar pain. I initially suspected a lumbar-spine disorder and promptly sought emergency medical care. Emergency-department clinicians detected discordant blood-pressure readings between my two arms and arranged urgent CT scanning, which identified acute aortic dissection. The medical team fully explained my critical clinical condition and coordinated urgent transfer to a specialized cardiovascular center for surgery. The entire treatment pathway was professional and standardized. My postoperative recovery was rapid, with complete resolution of leg numbness and weakness by the second postoperative day. At follow-up, I have regained full self-care capacity and independent mobility. I am deeply grateful to all clinical staff for timely diagnosis and life-saving treatment. My clinical experience reminds the public to attach importance to new-onset unexplained lower-limb numbness and weakness and to seek prompt medical assessment so as to avoid diagnostic delay for severe vascular disease.

## Abbreviations

AAD: Stanford type A aortic dissection
AD: Acute aortic dissection
CT: Computed tomography
CTA: Computed-tomography angiography
INR: International normalized ratio
mmHg: Millimetre of mercury
CPB: Cardiopulmonary bypass
MRI: Magnetic-resonance imaging
POD: Postoperative day
ED: Emergency department
