# Supplementary material for: Painless Stanford type A aortic dissection presenting with isolated bilateral lower-limb neurological deficits: a case report
Source: Egypt Heart J. 2026 Nov 13;78:86. doi: 10.1186/s43044-026-00799-4 (PMC13768529; doi:10.1186/s43044-026-00799-4)
Supplement: Supplementary file 1 [file 43044_2026_799_MOESM1_ESM.docx]

**Literature Comparison Table**

**Painless Stanford Type A Aortic Dissection Presenting with Isolated Bilateral Lower-Limb Neurological Deficits: A Case Report**

*Additional file 1 — Literature comparison table*

**1. Rationale**

This supplementary table systematically compares the present case with previously reported cases of painless aortic dissection (AD) or AD presenting predominantly with neurological manifestations. It substantiates the arguments developed in Section 3 (Literature comparison and clinical value of the case) of the main text and provides the additional material requested by Reviewer 4 regarding the adequacy of in-text citation of references [7]–[10].

The comparison encompasses publication details and reference numbering, study design, number of cases, patient demographics, Stanford classification, pain status, presenting or principal clinical manifestation, neurological findings, imaging modality, surgical strategy, neurological outcome, follow-up outcome, and anticoagulation regimen.

**2. Case-by-Case Comparison with Key Published Case Reports**

**Table S1. Item-by-item comparison of the present case with key published case reports.**

| **Variable** | **Present case** | **Ismail 2025 [4]** | **Hattab 2022 [5]** | **Campa 2023 [6]** | **Sakurai 2025 [7]** |
| --- | --- | --- | --- | --- | --- |
| Reference | — | [4] | [5] | [6] | [7] |
| Journal / Year | — | Cureus 2025;17(7):e87979 | Radiol Case Rep 2022;17(9):3294-3297 | Cureus 2023;15(1):e33389 | Cureus 2025;17(10):e94540 |
| PMID | — | 40666265 | 35846503 | 36618498 | 41246607 |
| Study design | Case report | Case report | Case report | Case report | Case report |
| No. of cases | 1 | 1 | 1 | 1 | 1 |
| Age / Sex | 58 y / Female | 59 y / Male | 66 y / Male | 42 y / Male | 84 y / Female |
| Stanford classification | Type A | Type A | Type A | Type A | Type A |
| Pain status | Completely painless | Painless | Completely asymptomatic / painless | Chest pain radiating to abdomen and back | Painless |
| Presenting / principal manifestation | Isolated bilateral lower-limb numbness and weakness | Syncope, loss of consciousness, right upper-limb weakness, slurred speech | Completely asymptomatic (chronic long-segmental dissection) | Sudden-onset paraplegia with sensory loss | Transient bilateral lower-limb weakness with bradycardia and hypotension |
| Neurological findings | Lower-limb motor power 3/5, diminished tendon reflexes, no pathological reflexes | Transient right upper-limb weakness, slurred speech | No neurological symptoms | Paraplegia with sensory loss (transverse myelopathy) | Transient bilateral lower-limb weakness |
| Associated signs | Interarm blood pressure difference (123/45 vs. 97/41 mmHg), distal pulses preserved | Shock (systolic BP 79 mmHg), left lower-limb ischaemia | Well-controlled hypertension | Heart rate 40 bpm, systolic BP 80 mmHg, dorsalis pedis pulses initially absent | Bradycardia, hypotension |
| Mechanism of spinal / peripheral ischaemia | Presumed transient spinal cord hypoperfusion | Multi-organ ischaemia | None | Spinal cord ischaemia (suspected Adamkiewicz artery involvement) | Spinal cord hypoperfusion secondary to systemic hypotension |
| Diagnostic imaging | Emergency non-contrast CT (double-lumen sign with inward displacement of calcified intimal plaque) → whole-aorta CTA | Bedside echocardiography plus CT | Transthoracic echocardiography plus CTA | Thoracoabdominal-pelvic CTA | Carotid ultrasound plus contrast-enhanced CT |
| Spinal MRI performed | No (declined by patient; no positive findings on CTA) | Not applicable | Not applicable | Ordered but not completed (declined by patient) | Not reported |
| Surgical procedure | Bentall procedure with total aortic arch replacement and frozen elephant trunk (mechanical composite valve graft) | Emergency aortic repair | Referred for surgery (details not specified) | Arch and Bentall repair | Valve-sparing supracoronary ascending aortic replacement |
| Neurological outcome | Complete recovery on POD 2 (5/5) | Good postoperative recovery | Not applicable | No recovery (persistent lower-limb dysfunction) | Complete postoperative recovery |
| Complications | No major complications | Not reported | Not reported | Complete heart block requiring pacemaker, renal failure, spinal cord ischaemia, pericardial effusion, sternal wound infection | None |
| Follow-up outcome | Complete recovery at 6 months, no recurrence | Good postoperative recovery | Referred for surgery | Persistent lower-limb dysfunction | Asymptomatic with no neurological deficit during follow-up |
| Anticoagulation | Lifelong warfarin after mechanical valve replacement (INR 2.0–3.0) | Not reported | Not reported | Not reported (after Bentall procedure) | Valve-sparing; anticoagulation / antiplatelet therapy not reported |

**3. Positioning Relative to Review Articles and Cohort Studies**

To further delineate the distinctive features of the present case, it was additionally compared with three larger-scale review or retrospective studies.

**Table S2. Positioning of the present case relative to review articles and cohort studies.**

| **Variable** | **Present case** | **Park 2004 [2] (IRAD)** | **Varela-Jaramillo 2024 [3]** | **Liu 2014 [10]** |
| --- | --- | --- | --- | --- |
| Study design | Case report | Registry analysis | Scoping review | Retrospective cohort |
| Sample size | 1 | 1,078 (IRAD) | 79 articles / 255 patients | 482 |
| Proportion of painless AD | Painless by definition (index case) | 6.4% | Up to 38% initially misdiagnosed at first presentation | 7.26% (35/482) |
| Pain status and prognosis | Painless with favourable recovery | Painless presentation associated with significantly increased in-hospital mortality | — | Painless group showed higher rates of hypotension and impaired consciousness |
| Distribution of neurological manifestations | Isolated bilateral lower-limb neurological deficit | — | Type A: motor syndrome 54.5%, impaired consciousness 43.8%; Type B: motor syndrome 57.0% (paraplegia 50.0%) | — |
| Proportion of Stanford type A | Type A | — | — | Significantly higher in the painless group than in the painful group (48.57% vs. 21.03%, P = 0.006) |
| Aortic arch involvement | Extensive arch involvement | — | — | Significantly more frequent in the painless group (19.23% vs. 5.52%, P = 0.019) |
| Diagnosis established at autopsy | No (diagnosed and treated antemortem) | — | 28% of cases diagnosed at autopsy | — |
| Incremental value of the present case | Complete absence of pain + isolated bilateral lower-limb neurological deficit + timely surgery + complete neurological recovery + favourable mid- to long-term outcome | Provides the mortality background of painless AAD | Provides a baseline distribution of neurological manifestations | Provides epidemiological and clinical characteristics of painless AD |

**4. Literature Support for the Surgical Strategy and Anticoagulation**

**Table S3. Literature support for the surgical strategy and anticoagulation regimen.**

| **Variable** | **Present case** | **Werner 2022 [8]** | **Norton 2019 [9]** |
| --- | --- | --- | --- |
| Study design | Case report | Long-term follow-up cohort | Review |
| Topic | Bentall procedure with total arch replacement and frozen elephant trunk | Long-term outcomes of the modified Bentall procedure with mechanical or biological composite valve grafts | Type A aortic dissection complicated by malperfusion syndrome |
| Key finding / supporting point | Composite valve graft (mechanical) provides definitive treatment of both root and arch pathology | Mechanical composite valve grafts demonstrate favourable long-term durability, supporting the choice of a mechanical prosthesis | Malperfusion is a leading cause of death and disability in type A aortic dissection and warrants prompt surgical revascularisation |
| Relevance to the present case | Mechanical composite valve graft Bentall procedure performed; lifelong postoperative anticoagulation | Provides evidence for the long-term safety of selecting a mechanical prosthesis | Provides evidence for timely surgical intervention in spinal cord and visceral malperfusion |

**5. PubMed Indexing Verification of Cited References**

**Table S4. PubMed indexing verification of the ten cited references.**

| **No.** | **Reference** | **Journal / Volume:Pages** | **PMID** | **PMCID** | **DOI** |
| --- | --- | --- | --- | --- | --- |
| [1] | Smedberg C, Hultgren R, Olsson C, Steuer J. Incidence, presentation and outcome of acute aortic dissection: results from a population-based study. | Open Heart 2024;11(1):e002595 | 38485121 | PMC10941176 | 10.1136/openhrt-2023-002595 |
| [2] | Park SW, Hutchison S, Mehta RH, et al. Association of painless acute aortic dissection with increased mortality. | Mayo Clin Proc 2004;79(10):1252-1257 | 15473405 | — | 10.4065/79.10.1252 |
| [3] | Varela-Jaramillo S, Taub-Krivoy A, Gómez-Gutiérrez MA, et al. Neurological manifestations of aortic dissection: a scoping review. | J Acute Med 2024;14(4):135-144 | 39624147 | PMC11608860 | 10.6705/j.jacme.202412_14(4).0001 |
| [4] | Ismail U. A case of Stanford type A aortic dissection presenting as syncope and neurologic deficits without pain: diagnostic pitfalls and a therapeutic dilemma. | Cureus 2025;17(7):e87979 | 40666265 | PMC12261810 | 10.7759/cureus.87979 |
| [5] | Hattab O, Amaqdouf S, Ouafi NE, Bazid Z. Painless long-segmental aortic dissection diagnosed by echocardiography: a case report of a diagnostic conundrum with potentially poor outcome. | Radiol Case Rep 2022;17(9):3294-3297 | 35846503 | PMC9284380 | 10.1016/j.radcr.2022.06.028 |
| [6] | Campa MR, Danboise B, Cambron JD. Sudden-onset paraplegia: a unique presentation of acute aortic dissection (AD). | Cureus 2023;15(1):e33389 | 36618498 | PMC9814824 | 10.7759/cureus.33389 |
| [7] | Sakurai K, Inoue R, Nishizawa T. Painless aortic dissection with bradycardia, hypotension, and lower limb weakness: a case report. | Cureus 2025;17(10):e94540 | 41246607 | PMC12612959 | 10.7759/cureus.94540 |
| [8] | Werner P, Gritsch J, Kaider A, et al. Long-term results of the modified Bentall procedure with mechanical and biological composite valve grafts. | Front Cardiovasc Med 2022;9:867732 | 35463795 | PMC9019133 | 10.3389/fcvm.2022.867732 |
| [9] | Norton EL, Khaja MS, Williams DM, Yang B. Type A aortic dissection complicated by malperfusion syndrome. | Curr Opin Cardiol 2019;34(6):610-615 | 31397690 | PMC7141757 | 10.1097/HCO.0000000000000667 |
| [10] | Liu ZY, Zou YL, Chai BL, Zeng HS. Analysis of clinical features of painless aortic dissection. | J Huazhong Univ Sci Technolog Med Sci 2014;34(4):582-585 | 25135731 | — | 10.1007/s11596-014-1319-8 |

Note: All ten references are retrievable in PubMed (https://pubmed.ncbi.nlm.nih.gov/) by PMID or DOI, in accordance with the journal’s requirement for reference retrievability.

**6. Summary of the Distinctive Features of the Present Case**

1. Complete absence of pain: the patient never experienced chest, back, abdominal, or lumbar pain throughout the clinical course, distinguishing this case from most published reports of “painless” dissection in which mild atypical symptoms such as syncope or impaired consciousness are nevertheless present.

2. Isolated bilateral lower-limb neurological deficit as the sole manifestation: there was no syncope, stroke, or impairment of consciousness, in clear contrast to the syncope, impaired consciousness, or hemiparesis commonly reported in references [3], [4], and [7].

3. Preservation of distal arterial pulses: this finding argues against peripheral arterial occlusion and supports transient spinal cord hypoperfusion rather than peripheral ischaemia, representing an important bedside discriminator.

4. Timely surgery with complete neurological recovery: the outcome contrasts sharply with the persistent lower-limb dysfunction reported in reference [6], suggesting that prompt and complete revascularisation can reverse transient spinal cord hypoperfusion.

5. Diagnostic value of indirect signs on non-contrast CT (inward displacement of calcified intimal plaque and the double-lumen sign): in resource-limited emergency settings, these findings facilitate early clinical suspicion and rapid triage.
