# Supplementary material for: Painless Stanford type A aortic dissection presenting with isolated bilateral lower-limb neurological deficits: a case report
Source: Egypt Heart J. 2026 Nov 13;78:86. doi: 10.1186/s43044-026-00799-4 (PMC13768529; doi:10.1186/s43044-026-00799-4)
Supplement: Supplementary file 2 [file 43044_2026_799_MOESM2_ESM.docx]

**出院用药清单**

**Discharge Medication List**

（Bentall 手术＋全主动脉弓置换＋冷冻象鼻支架植入术后 / After Bentall Procedure with Total Aortic Arch Replacement and Frozen Elephant Trunk Implantation）

患者姓名 / Patient name：____________________	性别 / Sex：女 / Female	年龄 / Age：58

诊断 / Diagnosis：无痛性 Stanford A 型主动脉夹层 / Painless Stanford Type A Aortic Dissection

出院日期 / Date of discharge：________年______月______日 （____ / ____ / ________）

**一、出院带药 / 1. Discharge Medications**

| **序号**  **No.** | **药物名称**  **Medication** | **规格**  **Strength** | **用法用量**  **Dose & Route** | **频次**  **Frequency** | **疗程**  **Duration** | **备注 / 注意事项**  **Notes / Precautions** |
| --- | --- | --- | --- | --- | --- | --- |
| 1 | 华法林钠片  Warfarin sodium | 2.5 mg/片  2.5 mg/tab | 口服，剂量按 INR 调整  PO., dose titrated to INR | 每日 1 次  Once daily | **长期服用（终生）**  **Long-term (lifelong)** | 机械瓣置换术后必需抗凝。目标 INR 2.0–3.0。出院后每 2–4 周复查 INR，稳定后可延长至每 4–12 周。避免剧烈改变饮食中维生素 K 摄入；注意出血征象（牙龈出血、皮下瘀斑、黑便、血尿），异常及时就诊。禁用/慎用与华法林相互作用的药物（如部分抗生素、NSAIDs、胺碘酮等），加用新药前请咨询医师。  Mandatory anticoagulation after mechanical valve replacement. Target INR 2.0–3.0. Check INR every 2–4 weeks initially, then every 4–12 weeks once stable. Avoid major fluctuations in dietary vitamin K. Watch for bleeding (gum bleeding, bruising, melena, hematuria). Consult a physician before adding any interacting drug (some antibiotics, NSAIDs, amiodarone, etc.). |
| 2 | 酒石酸美托洛尔片  Metoprolol tartrate | 25 mg/片  25 mg/tab | 口服  PO. | 每日 2 次  Twice daily | **长期服用**  **Long-term** | 控制心率（目标静息心率 60–80 次/分）与血压，降低主动脉壁剪切力，预防夹层进展与再发。不可自行停药或骤然减量。监测心率、血压；心动过缓（<55 次/分）或症状性低血压时就诊调整。  Rate control (target resting HR 60–80 bpm) and BP control to reduce aortic wall shear stress. Do not stop or abruptly reduce. Monitor HR and BP. |
| 3 | 缬沙坦胶囊  Valsartan | 80 mg/粒  80 mg/cap | 口服  PO. | 每日 1 次  Once daily | **长期服用**  **Long-term** | 控制血压（目标 <120/80 mmHg），延缓主动脉扩张。监测血压、肾功能与血钾；妊娠禁用。  Blood pressure control (target <120/80 mmHg). Monitor BP, renal function, and serum potassium. Contraindicated in pregnancy. |
| 4 | 阿托伐他汀钙片  Atorvastatin calcium | 20 mg/片  20 mg/tab | 口服，每晚  PO., at night | 每日 1 次  Once daily | **长期服用**  **Long-term** | 稳定动脉粥样硬化斑块，二级预防。监测肝功能与肌酸激酶；出现肌痛、乏力、深色尿及时就诊。  Plaque stabilization and secondary prevention. Monitor liver enzymes and CK; report myalgia, weakness, or dark urine. |
| 5 | 肠溶阿司匹林片  Aspirin (enteric-coated) | 100 mg/片  100 mg/tab | 口服，餐后  PO., after meal | 每日 1 次  Once daily | **长期服用**  **Long-term** | 抗血小板、动脉粥样硬化二级预防。与华法林联用增加出血风险，须在医师评估后使用并密切监测。有消化道溃疡史者加用胃黏膜保护剂。  Antiplatelet therapy. Combined with warfarin increases bleeding risk — use only under physician assessment with close monitoring. |
| 6 | 泮托拉唑肠溶片  Pantoprazole | 40 mg/片  40 mg/tab | 口服，早餐前  PO., before breakfast | 每日 1 次  Once daily | 视情况 1–3 个月  1–3 months as indicated | 胃黏膜保护，预防抗凝/抗血小板相关消化道出血。症状缓解后可遵医嘱停用。  Gastroprotection against anticoagulant/antiplatelet-related GI bleeding. May be discontinued as advised once symptoms resolve. |

**二、用药监测与随访重点 / 2. Monitoring and Follow-up**

• INR：出院后每 2–4 周复查 1 次，连续 2 次达标后可延长至每 4–12 周；调整剂量后 3–7 天复查。

INR: every 2–4 weeks after discharge; extend to every 4–12 weeks after two consecutive in-range results. Recheck 3–7 days after any dose change.

• 血压与心率：建议每日居家测量并记录，血压目标 <120/80 mmHg，静息心率 60–80 次/分。

BP and HR: home measurement and recording daily; target BP <120/80 mmHg, resting HR 60–80 bpm.

• 影像随访：术后 1、3、6、12 个月复查主动脉 CTA，此后每年 1 次，评估支架、人工血管及远端主动脉。

Imaging: aortic CTA at 1, 3, 6, and 12 months postoperatively, then annually, to assess the graft, stent, and distal aorta.

• 实验室：定期复查血常规、肝肾功能、凝血功能、血脂。

Laboratory: periodic CBC, liver and renal function, coagulation profile, and lipid panel.

• 康复：继续下肢功能与神经康复训练，循序渐进增加活动量。

Rehabilitation: continue lower-limb and neurological rehabilitation, with progressive activity.

**三、需立即就诊的情况 / 3. Seek Immediate Medical Attention If**

• 突发胸痛、背痛、腹痛或腰痛；

sudden chest, back, abdominal, or lumbar pain;

• 新发肢体麻木、无力、感觉异常，或大小便功能障碍；

new-onset limb numbness, weakness, sensory disturbance, or bladder/bowel dysfunction;

• 无法解释的出血（呕血、黑便、血尿、皮下大片瘀斑、牙龈或鼻出血不止）；

unexplained bleeding (hematemesis, melena, hematuria, extensive bruising, persistent gum or nasal bleeding);

• 头晕、晕厥、心悸、胸闷、呼吸困难；

dizziness, syncope, palpitations, chest tightness, or dyspnea;

• 发热、切口红肿渗液或其他感染征象。

fever, wound redness, swelling, discharge, or other signs of infection.

**四、其他说明 / 4. Additional Notes**

• 本清单所列华法林为机械瓣置换术后终生抗凝治疗，不可自行停药。

Warfarin in this list is lifelong anticoagulation therapy after mechanical valve replacement and must not be stopped without medical advice.

• 所有药物均须遵医嘱服用，不得自行增减剂量或更换药品；加用任何新药（包括中成药、保健品）前请咨询医师或药师。

All medications must be taken as prescribed; do not adjust doses or switch drugs on your own. Consult a physician or pharmacist before adding any new medication, including over-the-counter drugs and supplements.

• 外出就诊、拔牙、手术、接受有创操作前，须主动告知医师正在服用华法林及抗血小板药物。

Before any consultation, dental extraction, surgery, or invasive procedure, inform the physician that you are taking warfarin and an antiplatelet agent.

• 建议随身携带机械瓣卡及用药清单。

Carry your mechanical valve card and this medication list with you at all times.

| 开具医师 / Prescribing physician：____________________ | 签名 / Signature：____________________ |
| --- | --- |
| 科室 / Department：____________________ 联系电话 / Tel：____________________ | 日期 / Date：________年______月______日 |

*注：本清单为根据病例报告整理的出院用药模板，具体药物、剂量与疗程须以患者实际出院医嘱为准。*
